# Blunted Niacin Skin Flushing Response in Mood Disorders: A Meta‐Analysis of Case–Control Studies

**DOI:** 10.1155/da/1967324

**Published:** 2026-05-13

**Authors:** Qian Wang, Jinfeng Wang, Xiaowen Hu, Yan Gao, Shuhui Li, Tianqi Wang, Dandan Wang, Chunling Wan

**Affiliations:** ^1^ Bio-X Institutes, Key Laboratory for the Genetics of Developmental and Neuropsychiatric Disorders, Ministry of Education, Shanghai Jiao Tong University, Shanghai, China, sjtu.edu.cn; ^2^ Department of Gastrointestinal Surgery, Shanghai General Hospital, Shanghai Jiao Tong University School of Medicine, Shanghai, China, shsmu.edu.cn

**Keywords:** auxiliary diagnostic biomarker, meta-analysis, mood disorders, niacin skin flushing response, standardized niacin testing protocol

## Abstract

**Background:**

Mood disorders (MDs), including depressive disorder (DD) and bipolar disorder (BD), represent a major global health burden, yet the absence of objective biomarkers has persistently hindered their accurate clinical diagnosis. The niacin skin flushing response (NSFR) has shown promise as a potential biomarker; however, inconsistencies in diagnostic efficacy and response characteristics across studies have limited its utility. This meta‐analysis aims to systematically evaluate the NSFR in MD patients to address these discrepancies.

**Methods:**

A comprehensive search of PubMed, Embase, Cochrane Library, and Scopus databases was conducted for articles published through May 2025, identifying 18 eligible case–control studies involving 1848 participants (888 MD patients and 960 HC). Random‐effects models were used to assess the degree, speed, and sensitivity of NSFR, while subgroup analyses and meta‐regression explored potential sources of heterogeneity.

**Results:**

MD patients exhibited markedly blunted NSFR, characterized by reduced degree and speed of response (standardized mean difference [SMD] = −0.59, 95% confidence interval [CI]: [−1.12; −0.05] and SMD = 0.72, 95% CI: [0.35; 1.10]), while no statistically significant difference in NSFR sensitivity was observed (SMD = 0.51, 95% CI: [−0.18; 1.20]). Subgroup analyses identified substantial heterogeneity across detection methods, geographical regions, and control sources, highlighting the need for standardized protocols. Meta‐regression analyses indicated that sample size, publication year, gender distribution, and age did not significantly affect outcomes, further strengthening the robustness of our findings.

**Conclusion:**

This study reinforces the potential of NSFR as a biomarker for MD and highlights the critical need to address heterogeneity through standardized methodologies in future research to enhance its diagnostic reliability.

## 1. Introduction

Mood disorders (MDs), including depressive disorder (DD) and bipolar disorder (BD), has become a major global health burden due to their high prevalence and mortality [1]. Currently, the diagnosis of MD predominantly relies on structured interviews and psychometric evaluations [2], which are constrained by limited efficiency and insufficient objectivity. Despite extensive research, the underlying biological mechanisms of MD remain only partially elucidated, and the identification of reliable biomarkers continues to pose a critical challenge. The discovery of robust biomarkers has the potential to enhance diagnosis precision, enable personalized treatment strategies, and facilitate the stratification of patients into biologically homogeneous subgroups [3].

The niacin skin flushing response (NSFR) has emerged as a promising candidate biomarker for MD. NSFR is a physiological response caused by stimulating the skin surface with a solution of methyl nicotinate or oral administration of niacin, inducing vasodilation and resulting visible skin flushing. The phenomenon of insensitivity to niacin stimulation leading to attenuated NSFR, termed the blunted niacin response (BNR), was initially observed in patients with schizophrenia (SZ) in the 1980s [4]. Subsequent findings have suggested that BNR may serve as a potential biomarker for aiding in the diagnosis of SZ [5–11]. The molecular mechanism underlying the niacin flushing response has been well‐elucidated [9]. Niacin binds to G‐protein‐coupled receptors on epidermal cells, activating phospholipase A2 (PLA2), which hydrolyzes membrane phospholipids to release arachidonic acid (AA). Free AA is further oxidized by cyclooxygenase (COX) and subsequent downstream synthetases to produce prostaglandin (PG) D2 and PGE2. PGE2 and PGD2 induce capillary vasodilation and skin flushing. The PLA2–AA–COX pathway plays a central role in membrane lipid metabolism, inflammation, and oxidative stress [12, 13], making abnormal NSFR a significant implication for understanding the pathophysiology of MD.

In the early 21^st^ century, research on NSFR extended to MD, particularly DD and BD [6, 14–17]. Studies have reported that MD patients also exhibit BNR, albeit to a lesser extent than those with SZ [15, 17]. The BNR in DD and BD patients is characterized by reduced flushing degree and delayed flushing speed [7–9, 15, 17–23]. However, significant discrepancies exist in the reported prevalence of BNR in MD across studies [5, 6, 16, 24–28], which may be due to methodological limitations and small sample sizes, as well as differences in the subtypes of MD included in different studies. Initially, Hudson et al. [6] utilized oral niacin to monitor ear temperature changes and found no significant difference in NSFR between BD patients and healthy controls (HCs). This approach, however, was criticized for its lack of precision in assessing the flushing response. Ward et al. [29] introduced a more refined visual semi‐quantitative (VSQ) method, which involves applying filter paper soaked in aqueous methyl nicotinate (AMN) solutions to the forearm skin and scoring the erythema size relative to the stimulated area. This method has been widely adopted, with numerous studies demonstrating its utility in assessing BNR in both adult and adolescent MD patients [7, 8, 15, 17, 20], through few exceptions remain [24, 25, 27].

Advancements in quantitative assessment methods, such as laser Doppler flowmetry (LDF) and optical reflectance spectroscopy (ORS), have further enriched NSFR evaluation [5, 9, 16, 19, 26, 28]. However, these device‐dependent methods appear less effective than VSQ method in detecting BNR, potentially due to differences in measurement parameters. Only two out of six studies reported significant universality of BNR in MD using these methods [9, 19]. Recently, innovative artificial intelligence (AI) driven approaches have been developed to enhance NSFR testing by leveraging AI to identify erythema, improving accuracy, efficiency, and reducing human bias associated with VSQ. These AI‐driven methods have demonstrated excellent performance in detecting BNR in MD patients [21–23], highlighting their potential as reliable tools for NSFR assessment. Currently, diagnostic models based on various NSFR testing methods for distinguishing between MD and HC have shown promising results, with sensitivity ranging from 51% to 84% and specificity from 54% to 84% [8, 18, 21, 22, 25], underscoring the potential of NSFR as a noninvasive and rapid biomarker for MD.

Despite these encouraging findings, inconsistencies in study outcomes and methodologies highlight the need for a systematic evaluation of NSFR in MD. To address these gaps, this meta‐analysis aims to synthesize existing evidence on NSFR in MD patients. We systematically evaluated the degree, speed, and sensitivity of NSFR and explored potential sources of heterogeneity through subgroup analyses and meta‐regression, ultimately seeking to a comprehensive understanding of NSFR as a biomarker for MD. Furthermore, we aim to identify effective assessment methods and provide methodological considerations for future research and clinical applications.

## 2. Materials and Methods

### 2.1. Search Strategy

This meta‐analysis was conducted in accordance with the Preferred Reporting Items for Systematic Reviews and Meta‐Analyses (PRISMA) [30]. Relevant literature was searched in electronic databases, including PubMed, Embase, Cochrane Library, and Scopus, up to May 10, 2025. The following search terms were used: (niacin OR “nicotinic acid” OR nicotinate OR “methyl nicotinate” OR methylnicotinate OR “nicotinic acid methyl ester” OR “nicotinamide” OR “niacinamide” OR “ Vitamin B3") AND (depression OR “major depressive disorder” OR “depressive disorder” OR “depressive episode” OR “dysthymia” OR “depressive symptoms” OR “bipolar disorder” OR “mood disorders” OR “affective disorder"). No restrictions on language or publication dates were applied during the search process. In addition to electronic searches, we manually screened the reference lists of included studies. All identified studies were managed using EndNote software.

### 2.2. Eligibility Criteria

We first screened titles and abstracts, and then the full text to identify studies that met the following inclusion criteria: (1) case–control studies; (2) participants included patients with MD and HC; (3) exogenous niacin was added to the objects either orally or through topical application to the skin; (4) if the same dataset was published in two or more articles, we selected the article with the largest sample size or the most comprehensive data reporting; and (5) reporting of mean values and standard deviation (SD) in the study results. This rigorous selection process ensured methodological consistency and data comparability across included studies.

### 2.3. Data Extraction and Quality Assessment

The literature search, study selection, quality assessment, and data extraction were conducted independently by two researchers (Qian Wang and Jinfeng Wang) to ensure methodological rigor. Any disagreements were resolved through discussion between the two individuals, with adjudication by a third researcher (Dandan Wang) if disagreements persisted. The methodological quality of the studies was assessed using the Newcastle–Ottawa Scale (NOS) for observational studies [31]. The NOS employs a star‐based scoring system ranging from 0 to 9, where 0 stars indicate the lowest quality and 9 stars indicate the highest methodological quality. Studies achieving a score of ≥5 were considered to have sufficient methodological rigor [32] and were included in the final meta‐analysis [32]. For studies that passed quality assessment, the following essential data were systematically extracted: (1) first author, publication year, and country of origin; (2) sample size, age (years, mean), and gender distribution of MD patients and HCs, diagnostic criteria, source of control groups; (3) the detection methods and assessment indicators for NSFR; and (4) mean values and SD of the NSFR indicators for both MD and HC groups.

### 2.4. Statistical Analysis

Software R4.3.2 was used to conduct statistical analysis. To evaluate the differences in NSFR between the MD and HC groups, a random effect meta‐analysis was performed using meta package. The standardized mean differences (SMDs) with 95% confidence intervals (CIs) were calculated as the effect size for all included studies, with visualization by forest plots. Heterogeneity among the studies was evaluated using both the *Q* statistic and *I* ^2^ statistic [33], with *I* ^2^ values of 25%, 50%, and 75% indicating low, moderate, and high heterogeneity, respectively. Potential publication bias was assessed through visual inspection of funnel plots and quantified using Egger’s regression test [34]. Sensitivity analysis was conducted by examining the impact on the pooled effect estimates following systematic exclusion of each study individually. Mixed‐effects meta‐regression analysis and subgroup analyses were performed to investigate potential sources of heterogeneity. All tests were two‐tailed and statistical significance was defined as *p* < 0.05.

## 3. Results

### 3.1. Literature Search and Study Characteristics

**Figure 1:**
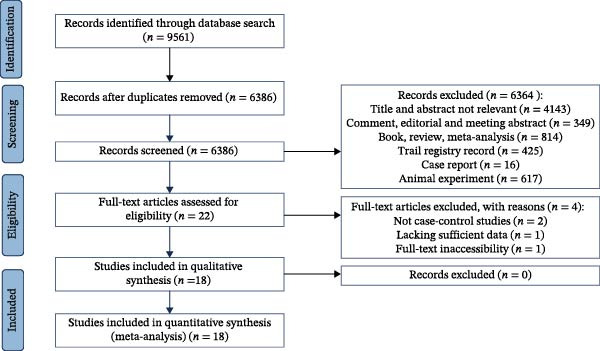
Flow chart of selection of studies for the meta‐analysis.

The electronic literature search identified a total of 9561 studies, with 3175 remaining after duplicates removal. Manual screening of reference lists did not yield additional eligible studies. Following a rigorous screening process based on titles and abstracts, 22 studies were selected for full‐text review. After applying the predefined inclusion criteria, four studies were excluded [14, 23, 35, 36], leaving 18 studies for quantitative synthesis [6–9, 15–22, 24–28, 5]. The detailed flowchart for the selection of studies is shown in Figure 1.

The characteristics of the 18 included case–control studies are summarized in Table 1. These studies enrolled a total of 1848 participants, including 888 patients diagnosed with MD and 960 HC. MD patients can be further categorized into 371 cases of DD, 438 cases of BD, 16 cases of psychotic MD (PMD), and 63 MD patients that lack a definitive classification. The studies spanned from 1997 to 2023, with geographical distribution as follows: China (9) [7, 8, 19–22, 25, 28, 5], the United Kingdom (3) [15–17], Germany (1) [26], the Netherlands (1) [24], Poland (1) [18], Canada (1) [6], Iran (1) [27], and the United States (1) (Table 1) [9]. All studies included participants of both genders. The assessment of NSFR evolved across studies. Following the initial study that characterized the NSFR by measuring ear temperature after oral niacin [6], eight studies employed the VSQ method [7, 8, 15, 17, 20, 24, 25, 27]. Subsequently, three studies utilized algorithms to automatically assess the flushing area [18, 21, 22], enhancing objectivity and precision. In addition to the flushing area, five studies evaluated changes in blood flow perfusion before and after AMN stimulation using LDF [9, 16, 19, 28, 5], while one study employed ORS to measure differences in reflection intensities [26].

**Table 1:** Characteristics of the case‐control studies included in the meta‐analysis.

Diagnosis	First author	Year	Country	Disease diagnostic criteria	Sample size		Gender (male/female)		Age (year, mean ± SEM)		Source of controls	Match (age and gender)	Assessment methods	NSFR manifestation	NOS score
					Case	Control	Case	Conrol	Case	Control					
Depressive disorder	Nianhong et al. [21]	2023	China	DSM‐5	38	47	13/25	20/27	14.5 ± 8.94	14.70 ±7.54	Students	Match	Automatic flushing area identification^a^	Blunted	6
	Wang et al. [22]	2023	China	ICD10	99	99	23/76	32/67	14.13 ± 1.42	14.38 ± 1.24	Social recruitment	Match	Automatic flushing area identification^a^	Blunted	7
	Ruihua et al. [19]	2021	China	DSM‐4	40	32	19/21	18/14	26.6 ± 8.6	33.7 ± 11.3	Community controls	Gender match	Laser Doppler flowmeter^b^	Blunted	7
	Wang et al. [8]	2021	China	ICD10	127	148	41/86	38/110	41.5 ± 16.1	34.7 ± 8.8	Hospital Staffs	Match	Visual semi‐quantitative ^c^	Blunted	6
	Smesny et al. [26]	2010	Germany	DSM‐4	30	30	15/15	15/15	46.2 ± 10	44.7 ± 8.4	Unknown	Match	Optical reflection spectrum^d^	No significant difference	6
	Bosveld‐van Haandel et al. [24]	2006	Netherlands	DSM‐4	17	16	1/16	8/8	40.2	35.9	Hospital recruitment	Not‐match	Visual semi‐quantitative^c^	No significant difference	7
	Ross et al. [17]	2004	England	DSM‐4	20	38	9/12	15/23	46 ± 13.42	42 ± 12.33	Unknown	Match	Visual semi‐quantitative^c^	Blunted	6
Bipolar disorder	Hu et al. [28]	2022	China	DSM‐4	41	80	24/17	27/53	26.61 ± 7.04	29.21 ± 4.83	Community controls	Match	Laser Doppler flowmeter^b^	Blunted	7
	Qing et al. [20]	2022	China	ICD10	23	48	6/17	32/16	14.39 ± 1.17	11.52 ± 4.33	Social recruitment	Not‐match	Visual semi‐quantitative^c^	Blunted	6
	Wang et al. [8]	2021	China	ICD10	179	148	115/64	38/110	37 ± 14.1	34.7 ± 8.8	Hospital staff	Match	Visual semi‐quantitative^c^	Blunted	6
	Karakula‐Juchnowicz et al. [18]	2020	Poland	DSM‐5	29	45	7/22	27/18	42	27	Unknown	Not‐match	Automatic flushing area identification^a^	Blunted	7
	Marouf et al. [27]	2016	Iran	DSM‐4	25	25	8/17	11/13	34 ± 10.9	36.1 ± 10.8	Unknown	Match	Visual semi‐quantitative^c^	No significant difference	6
	Yao et al. [9]	2016	Americian	DSM‐4	59	87	31/28	40/47	46.36 ± 8.36	36.41 ± 18.09	Community controls	Gender match	Laser Doppler flowmeter^b^	No significant difference	7
	Liu et al. [25]	2007	China	DSM‐4	18	40	8/10	17/23	29.2 ± 11.4	29.3 ± 9.3	Hospital Staffs	Match	Visual semi‐quantitative^c^	No significant difference	6
	Ross et al. [16]	2004	England	DSM‐4	26	32	13/13	17/14	42 ± 15.3	37 ± 11.31	Advertising recruitment	Match	Laser Doppler flowmeter^b^	No significant difference	6
	Maclean et al. [15]	2003	England	DSM‐4	20	34	12/8	14/21	41.5 ± 12.4	34.4 ± 10	Social and hospital recruitment	Gender match	Visual semi‐quantitative^c^	Blunted	6
	Hudson et al. [6]	1997	Canada	DSM‐3	18	28	4/14	14/14	37.95 ± 11.46	36.71 ± 10.27	Advertising recruitment	Age match	Oral niacin^e^	No significant difference	7
Psychotic mood disorder	Gan et al. [5]	2022	China	DSM‐4	16	68	4/12	27/41	29.8 ± 7.31	30.65 ± 11.36	Community controls	Gender match	Laser Doppler flowmeter^b^	No significant difference	7
Mood disorder	Sun et al. [7]	2017	China	ICD10	63	63	34/29	10/53	37.97 ± 1.75	59.57 ± 1.26	Hospital Staffs	Not‐match	Visual semi‐quantitative^c^	Blunted	6

Abbreviations: NOS, Newcastle–Ottawa scale; NSFR, niacin skin flushing response; SEM, standard error of the mean.

^a^Automatic flushing area identification: aqueous methyl nicotinate (AMN) was applied to the forearm skin, and the erythema area was automatically calculated using image processing algorithms.

^b^Laser Doppler flowmeter: AMN was applied to the forearm skin, and alterations in local blood perfusion were assessed before and after stimulation.

^c^Visual semi‐quantitative: AMN was applied to the forearm skin, and erythema size was manually scored relative to the stimulated area.

^d^Optical reflection spectrum: AMN was applied to the forearm skin, and the changes in reflectance intensity were measured before and after stimulation.

^e^Oral niacin: changes in ear temperature were monitored after oral niacin.

The NOS scale was employed to assess the methodological quality of the included articles. As detailed in Table 1 and Supporting Information 1: Table S1, all studies achieved NOS scores of ≥6, indicating moderate to high methodological quality. With the exception of the 1997 study [6], all other studies accounted for potential confounding factors such as gender and age in the analysis of flushing response. Regarding HC group selection, eight studies recruited HC from community or social settings [6, 9, 16, 19, 20, 22, 28], while the remaining studies utilized specific populations (e.g., hospital staff or students) [7, 8, 15, 21, 24, 25] or did not explicitly report the HC sources [17, 18, 26, 27].

### 3.2. Attenuated Degree of NSFR in MD

**Figure 2:**
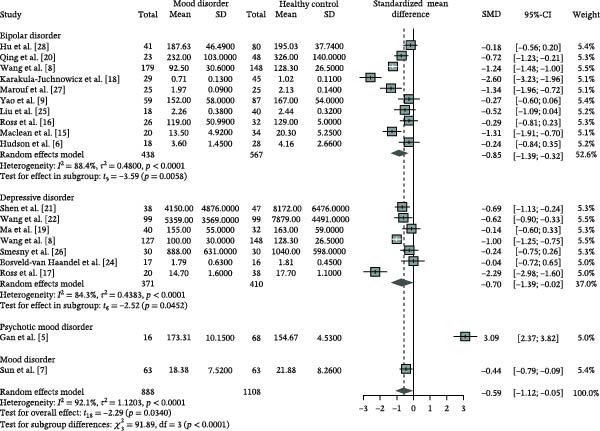
Forest plot of the degree of the niacin skin flushing response in mood disorders compared to healthy controls using a random‐effects model. Gan et al. [5] was later identified as an outlier based on the Baujat plot and excluded from subsequent analyses. CI, confidence interval; SD, standard deviation; SMD, standardized mean difference.

All 18 included studies provided quantitative data on NSFR degree (Supporting Information 1: Table S2). Notably, one study contributed separate data for both DD and BP groups and was counted twice in the analysis [8]. The results reveal a significant reduction in NSFR degree among MD patients compared to HCs (Figure 2, SMD = −0.59, 95% CI: [−1.12; −0.05]). This attenuation was consistently observed in both DD and BD patients, the two major MD subgroups (DD, SMD = −0.70, 95% CI: [−1.39; −0.02]; BD, SMD = −0.85, 95% CI: [−1.39; −0.32]). The *Q* values and *I* ^2^ statistics revealed high heterogeneity among the studies, regardless of whether considering the entire MD group or the DD and BD subgroups (MD, *Q* = 226.56, df = 18, *p* < 0.0001, *I* ^2^ = 92.1%; DD, *Q* = 38.34, df = 6, *p* < 0.0001, *I* ^2^ = 84.3%; BD, *Q* = 77.76, df = 9, *p* < 0.0001, *I* ^2^ = 88.4%). The Baujat plot identified that Gan et al. [5] as an potential outlier (Supporting Information 1: Figure S1), which had the most significant impact on overall heterogeneity and results. The outlier status of Gan et al. [5] may be attributed to its participants being PMD patients, which may have different NSFR patterns compared to other studies with DD and MD as the main participants. Moreover, the study included only 16 PMD patients, increasing susceptibility to confounding factors and limiting its representativeness of the PMD subgroup. Consequently, Gan et al. [5] was excluded from the final analysis.

After excluding Gan et al. [5], the effect size of NSFR degree increased (Supporting Information 1: Figure S2, SMD = −0.77, 95% CI: [−1.12; −0.42]), while heterogeneity decreased (*I* ^2^ = 85.9%). Despite this improvement, substantial heterogeneity remained (*Q* = 120.21, df = 17, *p* < 0.0001, *I* ^2^ = 85.9%). No significant publication bias was observed using funnel plots and Egger’s linear regression (Supporting Information 1: Figure S3, *t* = 0.05, df = 16, *p* = 0.9637). Furthermore, the leave‐one‐out sensitivity analysis demonstrated the robustness of the findings, as the exclusion of any single study did not alter the significance of the observed effect (Supporting Information 1: Figure S4). Collectively, these results highlight a significant attenuation of the NSFR degree in MD patients, with consistent patterns observed in both DD and BD subgroups.

### 3.3. Reduced Speed but Unaltered Sensitivity of the NSFR in MD

**Figure 3:**
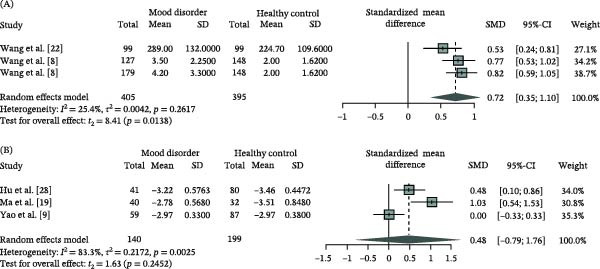
Forest plot of the speed (A) and sensitivity (B) of the niacin skin flushing response in mood disorders compared to healthy controls using a random‐effects model. CI, confidence interval; SD, standard deviation; SMD, standardized mean difference.

The speed of NSFR, quantified by the EC50—the duration required to achieve half of the maximum flushing area—was evaluated in three studies (Supporting Information 1: Table S2). A lower EC50 value indicates a faster flushing response. The analysis revealed a significant reduction in response speed among MD patients compared to HC (Figure 3A, SMD = 0.72, 95% CI: [0.35; 1.10]). The included studies demonstrated low and statistically insignificant heterogeneity (*Q* = 2.68, df = 2, *p* = 0.2617, *I* ^2^ = 25.4%), supporting the consistency of this finding.

Three studies employing the LDF method reported the sensitivity of NSFR, expressed as EC50 or lgEC50 values, which quantifies the niacin concentration required to elicit half of the maximum blood flow response (Supporting Information 1: Table S2). No statistically significant difference in sensitivity was observed between MD patients and HC (Figure 3B, SMD = 0.48, 95% CI: [−0.79; 1.76]). However, the *Q* value and *I* ^2^ statistics revealed high heterogeneity among the studies (*Q* = 12.00, df = 2, *p* = 0.0025, *I* ^2^ = 83.3%), indicating that the current evidence is limited. Due to the limited number of studies investigating the NSFR speed and sensitivity parameters, assessment of publication bias was not conducted [37]. This limitation should be considered when interpreting these findings. Collectively, the available evidence supports blunted NSFR in MD patients, particularly in terms of response degree and speed, whereas the evidence regarding sensitivity remains limited and inconclusive.

### 3.4. Subgroup Analysis and Meta‐Regressions of the NSFR in MDs

The attenuation of NSFR highlights its potential as a biomarker. However, the substantial heterogeneity across studies restricts its clinical utility. To address this, we performed subgroup analyses and meta‐regression to explore the potential sources of heterogeneity. The assessment of NSFR degree in MD patients compared to HC revealed significant variations based on detection methods and control sources (Table 2 and Figure 4A,B; *χ* ^2^ = 15.32, df = 4, *p* = 0.0041; *χ* ^2^ = 9.60, df = 3, *p* = 0.0233). Among the three primary detection methods, the VSQ method (SMD = −0.98, 95% CI: [−1.46; −0.49]) and LDF method (SMD = −0.22, 95% CI: [−0.33; −0.12]) demonstrated statistically significant effect sizes, with the former showing a notably larger effect. The automated flushing area identification method yielded the highest but not significant effect size (SMD = −1.28, 95% CI: [−4.04; 1.49]). High heterogeneity was observed in studies using the VSQ method (*I* ^2^ = 80.3%, *p* < 0.0001) and the automated flushing area identification method (*I* ^2^ = 93.8%, *p* < 0.0001), while studies employing LDF method showed no heterogeneity (*I* ^2^ = 0%, *p* = 0.9591).

Figure 4Subgroup analysis of the degree of niacin skin flushing response in mood disorders compared to healthy controls using a random‐effects model. (A) detection methods, (B) control sources, (C) age groups, and (D) geographical regions. CI, confidence interval; SD, standard deviation; SMD, standardized mean difference.

**Figure:**
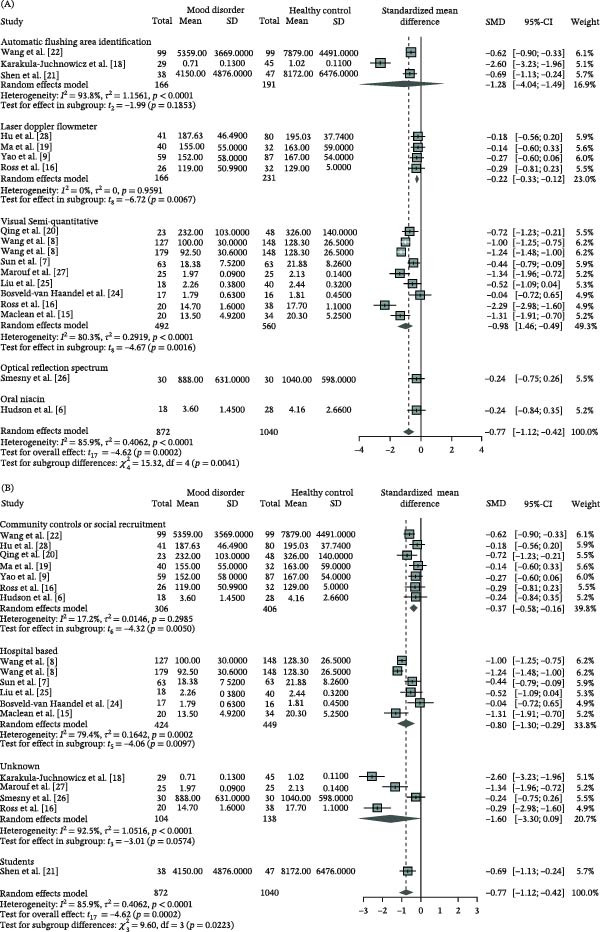

**Figure:**
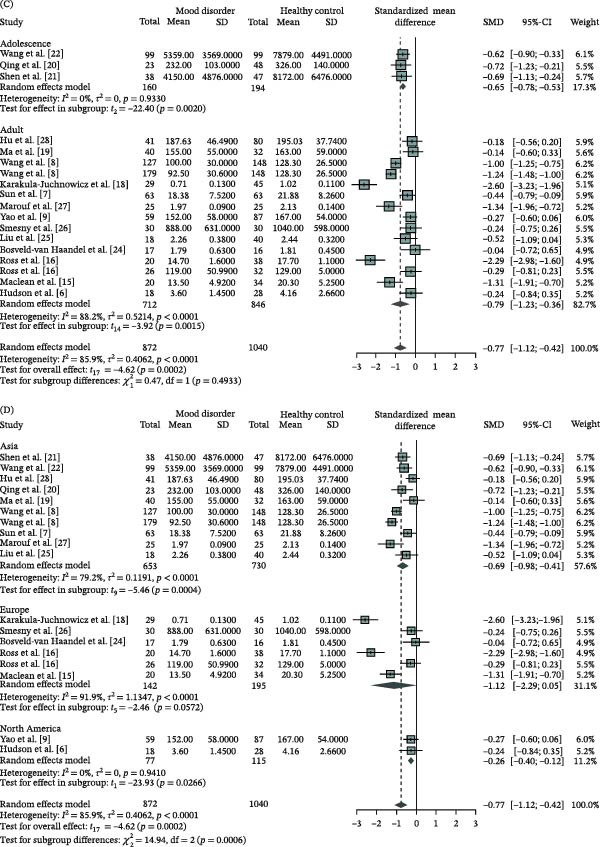

**Table 2:** Subgroup analysis of the niacin skin flushing response in mood disorders.

Stratification group	*N*	SMD	[95% CI]	Weight (%)	Heterogeneity test		Subgroup differences	
					*I* ^2^ (%)	*p*‐Value	*χ* ^2^	*p*‐Value
Detection methods							15.32	0.0041
Visual semi‐quantitative	9	−0.98	[−1.46; −0.49]	49.3	80.30	<0.0001	—	—
Laser Doppler flowmeter	4	−0.22	[−0.33; −0.12]	23.0	0	0.9591		
Automatic flushing area identification	3	−1.28	[−4.04; 1.49]	16.9	93.80	<0.0001		
Optical reflection spectrum	1	−0.24	[−0.75; 0.26]	5.5	—	—		
Oral niacin	1	−0.24	[−0.84; 0.35]	5.2	—	—		
Control sources							9.6	0.0233
Community controls or social recruitment	7	−0.37	[−0.58; −0.16]	39.80	17.20	0.2985	—	—
Hospital based	6	−0.8	[−1.30; −0.29]	33.80	79.40	0.0002		
Unknown	4	−1.6	[−3.30; 0.09]	20.70	92.50	<0.0001		
Students	1	−0.69	[−1.13; −0.24]	5.70	—	—		
Age							0.47	0.4933
Adult	15	−0.79	[−1.23; −0.36]	82.70	88.20	<0.0001	—	—
Adolescence	3	−0.65	[−0.78; −0.53]	17.30	0	0.933		
Geographical regions							14.94	0.0006
Asia	10	−0.69	[−0.98; −0.41]	57.60	79.20	<0.0001	—	—
Europe	6	−1.12	[−2.29; 0.05]	31.10	91.90	<0.0001		
North America	2	−0.26	[−0.40; −0.12]	11.20	0	0.941		

Abbreviations: CI, confidence intervals; NSFR, niacin skin flushing response; SMD, standardized mean differences.

Regarding control sources (Table 2 and Figure 4B), significant effect sizes were observed in studies with controls recruited from the community or social settings (SMD = −0.37, 95% CI: [−0.58; −0.16]) and hospitals (SMD = −0.80, 95% CI: [−1.30; −0.29]), with studies using unspecified control sources showing a trend toward significance (SMD = −1.60, 95% CI: [−3.30; 0.09]). Low heterogeneity was found in studies with controls from the community or social recruitment (*I* ^2^ = 17.2%, *p* = 0.2985), whereas high heterogeneity was present in studies with hospital‐based controls (*I* ^2^ = 79.4%, *p* = 0.0002) and those with unspecified control sources (*I* ^2^ = 92.5%, *p* < 0.0001).

The subgroup analysis based on patient age revealed that both adult and adolescent MD patients showed significantly attenuated NSFR degree compared to HC, with no significant difference between the two age groups (Table 2 and Figure 4C, *χ* ^2^ = 0.47, df = 3, *p* = 0.4933). The effect size for adult MD patients (SMD = −0.79, 95% CI: [−1.23; −0.36]) was slightly higher than that for adolescent MD patients (SMD = −0.65, 95% CI: [−0.78; −0.53]). Studies involving adolescent MD patients demonstrated no heterogeneity (*I* ^2^ = 0%, *p* = 0.9330), whereas those focusing on adult MD patients exhibited high heterogeneity (*I* ^2^ = 88.2%, *p* < 0.0001), potentially due to variations in study design or methodological approaches.

Geographical subgroup analysis revealed significant regional variations in NSFR attenuation among MD patients (Table 2 and Figure 4D; *χ* ^2^ = 14.94, df = 2, *p* = 0.0006). The most pronounced attenuation was observed in Asian cohorts (SMD = −0.69; 95% CI: −0.98 to −0.41; *p* < 0.001), followed by North American (SMD = −0.26; 95% CI: −0.40 to −0.12; *p* = 0.001) and European cohorts (SMD = −1.12; 95% CI: −2.29 to 0.05; *p* = 0.06). High heterogeneity was noted in studies conducted in Asia (*I* ^2^ = 79.2%, *p* < 0.0001) and Europe (*I* ^2^ = 91.9%, *p*  < 0.0001), while studies from North America showed no heterogeneity (*I^2^* = 0%, *p* = 0.9410).

**Figure 5:**
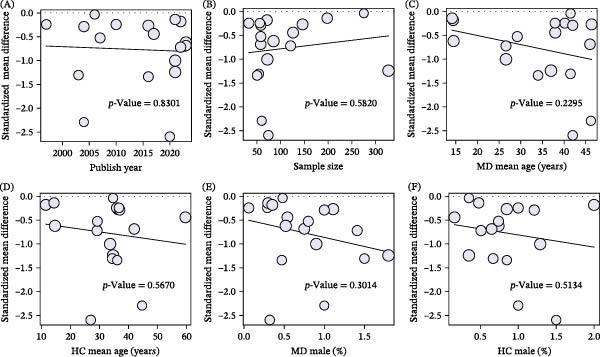
Meta‐regression analysis of the degree of the niacin skin flushing response in mood disorders (MD) compared to healthy controls (HC). (A) Sample size. (B) Publish year. (C) MD mean age. (D) HC mean age. (E) MD male (%). (F) HC male (%).

Meta‐regression analyses further indicated that sample size, publication year, male proportion, and age did not significantly influence the effect sizes of NSFR between MD patients and HC (Figure 5). These results suggest that these factors are not major contributors to the observed heterogeneity in effect sizes. In summary, the aforementioned findings underscore that the methodological factors, including detection methods and control sources, constitute the primary sources of heterogeneity on NSFR assessment in MD patients, highlighting the critical need for standardized protocols to improve the consistency and reliability of NSFR‐based assessments in MDs.

## 4. Discussion

This meta‐analysis provides robust evidence that NSFR is significantly attenuated and delayed in MD patients, including DD and BD, supporting its potential as a diagnostic biomarker. The VSQ method has emerged as the most effective assessment method for identifying NSFR blunting in MD, while AI‐based automated methods show considerable promise for future applications. Subgroup analyses highlighted the influence of methodological, geographical, and control source factors on study outcomes, underscoring the need for standardized protocols. The BNR may reflect dysregulation within the AA/COX pathway, and the meta‐analytic findings provide a solid foundation for further investigation into the biological mechanisms and clinical applications of NSFR in MD. Additionally, despite the distinct clinical classifications of DD and BD as MD subgroups, their similar BNR patterns suggest the existence of biologically homogeneous subpopulations within both disease categories.

### 4.1. Methodological Considerations for NSFR as a Diagnostic Biomarker for MDs

The BNR observed in MD patients has driven the development of NSFR‐based diagnostic models to differentiate MD from HC, with NSFR‐based models showing moderate sensitivity (51%–84%) and specificity (54%–84%) [8, 18, 21, 22, 25]. The age‐based subgroup analysis and meta‐regression results revealed that NSFR blunting was consistent across age groups, supporting its utility as a reliable biomarker for MD, even in early disease stages [20–22]. This finding is particularly significant, especially for adolescent MD, a population often characterized by atypical symptom presentation and increased diagnostic challenges compared to adult MD [38, 39]. Adolescence represents a diagnostically challenging period due to overlapping MD symptoms with normative emotional fluctuations. The relatively consistent NSFR alteration observed in adolescents may, therefore, provide complementary biological information for early identification of individuals at risk. If validated in larger longitudinal studies, NSFR may serve as a potential physiological marker to support early detection and guide timely intervention in this population. Furthermore, the observed inverse correlation between NSFR severity and depressive symptoms further suggests its potential as a prognostic indicator [26].

However, the substantial variability in reported BNR prevalence among MD populations hindered its clinical application. This meta‐analysis robustly validates the diagnostic potential of BNR and identifies key heterogeneity sources through subgroup analysis, providing actionable insights for clinical optimization. The high heterogeneity across studies (*I* ^2^ = 85.9%) underscores the influence of methodological and demographic factors on NSFR outcomes. The VSQ method demonstrated larger effect sizes than device‐based approaches, including LDF and ORS, suggesting greater sensitivity in detecting BNR. This difference may partly reflect variations in measurement characteristics. VSQ captures the integrated visible vascular response across the skin surface, whereas device‐based techniques typically quantify localized physiological changes in blood flow. However, the reliance of VSQ on subjective scoring introduces observer bias, prompting the development of automated flushing area identification techniques. Although these automated methods yielded the highest effect size, their results were not statistically significant due to limited studies and methodological variability. Nevertheless, their potential to standardize and enhance NSFR assessment accuracy makes them a promising avenue for future research.

Another methodological consideration is the lack of standardized protocols for niacin concentration and response time across studies. As summarized in Supporting Information 1: Table S2, substantial variability exists in these parameters, which complicates cross‐study comparisons and may hinder clinical translation. Future research should aim to establish standardized stimulation and observation conditions. Moreover, given that single‐concentration NSFR indicators have demonstrated good discriminative performance in diagnostic modeling [18, 22], simplified protocols based on optimized single‐time‐point measurements with shorter observation periods may improve testing efficiency and facilitate large‐scale screening applications.

The source of HCs significantly influenced NSFR outcomes. Studies with community‐based controls, which better represent the general population, exhibited low heterogeneity and significant effect sizes, whereas those with hospital‐based or unspecified controls showed high heterogeneity. These findings underscore the necessity of recruiting community‐based controls in future studies to ensure population representativeness, mitigate potential biases arising from atypical demographic or clinical characteristics inherent. In the geographic subgroup analysis, significant NSFR blunting was observed in Asian and North American cohorts, whereas the pooled effect for European cohorts approached but did not reach statistical significance (*p* = 0.0572). This finding may reflect the limited number of studies or smaller sample sizes in the European subgroup rather than true biological differences. Further research with more diverse geographic representation is needed to confirm the consistency of NSFR alterations across populations.

### 4.2. Pathological Implications for the NSFR Blunting in MDs

Both DD and BD patients exhibit comparable BNR, a phenomenon also extensively documented in SZ [5–11], suggesting that NSFR may serve as a transdiagnostic physiological marker reflecting shared biological processes rather than as a disorder‐specific diagnostic tool. The NSFR is mediated by the PLA2–AA–COX2 cascade, which generates PGs responsible for vasodilation and skin flushing [9]. Dysregulation in any link of this pathway can lead to abnormal niacin flushing responses. First, disrupted membrane lipid homeostasis and AA deficiency have been reported in both MD and SZ [40–42], resulting in substrate insufficiency for the pathway. Emerging evidence further demonstrates a significant association between reduced polyunsaturated fatty acids (PUFAs) and BNR in DD [41]. Increased expression and activity of PLA2 and COX2 have been reported in MD [43–47], leading to excessive hydrolysis of membrane PUFAs and contributing to BNR. Additionally, this pathway is highly sensitive to inflammatory processes and oxidative stress [12], both of which play crucial roles in MD pathophysiology. Chronic low‐grade inflammation, increased lipid peroxidation, and reduced antioxidant capacity impair synaptic signaling and neuroplasticity [48–51], potentially driving core symptoms such as anhedonia, emotional dysregulation, and cognitive deficits [48].

In summary, persistent inflammation and oxidative stress likely drive overactivation of the AA/COX pathway in MDs, resulting in lipid peroxidation and PUFA depletion, which manifest as BNR. Moreover, alterations in vasomotor regulation or microvascular function may also contribute to the blunted flushing response [52, 53]. These vascular factors may influence cutaneous blood flow responses to niacin stimulation and warrant further investigation. Collectively, patients exhibiting BNR may represent a biologically homogeneous subgroup of MD characterized by these shared pathological mechanisms. These findings not only provide novel insights into the etiology of MD but also suggest promising personalized therapeutic strategies, such as targeted PUFA supplementation.

### 4.3. Limitations

This meta‐analysis has several limitations. First, the high heterogeneity observed across studies, despite subgroup analyses, suggests that unaccounted factors, such as medication status and illness duration, may influence NSFR outcomes. Second, many of the included studies had relatively small sample sizes, which may affect the stability and generalizability of the findings. Third, the limited number of studies employing automated flushing area identification restricts the ability to draw definitive conclusions about their efficacy. Fourth, most included studies were conducted in Asian and European populations, and the limited representation of other regions may restrict the generalizability of the findings.

## 5. Conclusion

This meta‐analysis provides robust evidence that NSFR is significantly attenuated in MD patients, including DD and BD, supporting its potential as a reliable diagnostic biomarker for MDs. However, the substantial heterogeneity observed across studies underscores the critical need for standardized assessment protocols, including the adoption of automated methods to minimize observer bias and enhance precision. Future research should focus on elucidating the biological mechanisms underlying NSFR attenuation and exploring its potential for targeted interventions in MD. Addressing these challenges is essential for translating NSFR into clinical practice, enabling its integration into personalized diagnostic and therapeutic strategies.

## Author Contributions

**Qian Wang:** conceptualization, formal analysis, investigation, data curation, methodology, validation, visualization, writing – original draft, writing – review and editing. **Jinfeng Wang:** investigation, data curation, methodology, validation. **Xiaowen Hu:** writing – review and editing, supervision. **Yan Gao, Shuhui Li, and Tianqi Wang:** investigation. **Dandan Wang:** conceptualization, data curation, writing – review and editing, supervision, project administration. **Chunling Wan:** conceptualization, methodology, funding acquisition, project administration, resources, supervision, writing – review and editing. All authors contributed to the interpretation of the results.

## Funding

This work was supported by grants from the National Natural Science Foundation of China (Grant 82471522), the STI2030‐Major Projects (Grant 2021ZD0200800), and the Natural Science Foundation of Shanghai (Grant 23ZR1433300).

## Disclosure

All authors have approved the published version of the manuscript.

## Conflicts of Interest

The authors declare no conflicts of interest.

## Supporting Information

Additional supporting information can be found online in the Supporting Information section.

## Supporting information


**Supporting Information 1** Table S1. The study quality scores of the studies included in meta‐analysis. Table S2. Definitions and acquisition methods of niacin skin flushing response (NSFR) measures included in the meta‐analysis. Figure S1. The Baujat plot of the degree of the niacin skin flushing response in mood disorders compared to healthy controls. Figure S2. Forest plot of the degree of the niacin skin flushing response in mood disorders compared to healthy controls (excluding Gan et al. [5]). Figure S3. Funnel plots for identifying publication bias in the meta‐analysis of the degree of the niacin skin flushing response. Figure S4. The leave‐one‐out sensitivity analysis of the degree of the niacin skin flushing response.

**Supporting Information 2** The PRISMA 2020 checklist.

## Data Availability

The datasets generated and/or analyzed during the current study are available from the corresponding author upon reasonable request.
