# Supplementary material for: Blunted Niacin Skin Flushing Response in Mood Disorders: A Meta‐Analysis of Case–Control Studies
Source: Depress Anxiety. 2026 May 13;2026:1967324. doi: 10.1155/da/1967324 (PMC13169500; doi:10.1155/da/1967324)
Supplement: Supplementary file 1 — Supporting Information 1 Table S1. The study quality scores of the studies included in meta‐analysis. Table S2. Definitions and acquisition methods of niacin skin flushing response (NSFR) measures included in the meta‐analysis. Figure S1. The Baujat plot of the degree of the niacin skin flushing response in mood disorders compared to healthy controls. Figure S2. Forest plot of the degree of the niacin skin flushing response in mood disorders compared to healthy controls (excluding Gan et al. [5]). Figure S3. Funnel plots for identifying publication bias in the meta‐analysis of the degree of the niacin skin flushing response. Figure S4. The leave‐one‐out sensitivity analysis of the degree of the niacin skin flushing response. [file DA-2026-1967324-s002.docx]

**Supplementary Materials**

**Blunted Niacin Skin Flushing Response in Mood Disorders: A Meta-analysis of Case-Control Studies**

| **Supplementary Table 1.** The study quality scores of the studies included in meta-analysis |
| --- |
| **Supplementary Table 2.** Definitions and acquisition methods of niacin skin flushing response (NSFR) measures included in the meta-analysis. |
| **Supplementary Figure 1.** The Baujat plot of the degree of the niacin skin flushing response in mood disorders compared to healthy controls. |
| **Supplementary Figure 2.** Forest plot of the degree of the niacin skin flushing response in mood disorders compared to healthy controls (Excluding Gan 2022). |
| **Supplementary Figure 3.** Funnel plots for identifying publication bias in the meta-analysis of the degree of the niacin skin flushing response. |
| **Supplementary Figure 4.** The leave-one-out sensitivity analysis of the degree of the niacin skin flushing response. |

**Supplementary Table 1. The study quality scores of the studies included in meta-analysis**

| **Reference (first author year)** | **Selection** | | | | **Comparability** | **Exposure** | | | **Total Score** |
| --- | --- | --- | --- | --- | --- | --- | --- | --- | --- |
|  | Is the case definition adequate? | Representativeness of the cases | Selection of Controls | Definition of Controls | Comparability of cases and controls on the basis of the design or analysis | Ascertainment of exposure | Same method of ascertainment for cases and controls | Non-Response rate |  |
| **Shen 2023** [[1](#_ENREF_1)] | * | * | 0 | * | * | 0 | * | * | 6 |
| **Wang 2023** [[2](#_ENREF_2)] | * | * | * | * | * | 0 | * | * | 7 |
| **Gan 2022** [[3](#_ENREF_3)] | * | * | * | * | * | 0 | * | * | 7 |
| **Hu 2022** [[4](#_ENREF_4)] | * | * | * | * | * | 0 | * | * | 7 |
| **Qing 2022** [[5](#_ENREF_5)] | * | * | 0 | * | * | 0 | * | * | 6 |
| **Ma 2021** [[6](#_ENREF_6)] | * | * | * | * | * | 0 | * | * | 7 |
| **Wang 2021** [[7](#_ENREF_7)] | * | * | 0 | * | * | 0 | * | * | 6 |
| **Karakula-Juchnowicz 2020** [[8](#_ENREF_8)] | * | * | 0 | * | * | * | * | * | 7 |
| **Sun 2017** [[9](#_ENREF_9)] | * | * | 0 | * | * | 0 | * | * | 6 |
| **Marouf 2016** [[10](#_ENREF_10)] | * | * | 0 | * | * | 0 | * | * | 6 |
| **Yao 2016** [[11](#_ENREF_11)] | * | * | * | * | * | 0 | * | * | 7 |
| **Smesny 2010** [[12](#_ENREF_12)] | * | * | 0 | * | * | 0 | * | * | 6 |
| **Liu 2007** [[13](#_ENREF_13)] | * | * | 0 | * | * | 0 | * | * | 6 |
| **Bosveld-van 2006** [[14](#_ENREF_14)] | * | * | 0 | * | * | * | * | * | 7 |
| **Ross 2004** [[15](#_ENREF_15)] | * | * | 0 | * | * | 0 | * | * | 6 |
| **Ross 2004** [[16](#_ENREF_16)] | * | * | 0 | * | * | 0 | * | * | 6 |
| **Maclean 2003** [[17](#_ENREF_17)] | * | * | 0 | * | * | 0 | * | * | 6 |
| **J.Hudson 1997** [[18](#_ENREF_18)] | * | * | * | * | 0 | * | * | * | 7 |

**Supplementary Table 2. Definitions and acquisition methods of niacin skin flushing response (NSFR) measures included in the meta-analysis.**

| NSFR Measure | Study | Assessment Method | Niacin Concentrations | Response Time | Observation Time | Quantitative Indicator | Definition and Acquisition Method |
| --- | --- | --- | --- | --- | --- | --- | --- |
| Degree | Wang 2023[[2](#_ENREF_2)] | Automatic Flushing Area Identification | 6 concentrations (60, 20, 6.67, 2.22, 0.74, 0.25 mM) | 1min | 10 min  (every 10s) | Total Area | The total area of the flushing area over 10 min across all six niacin concentrations, calculated by an automated algorithm. |
| Degree | Shen 2023[[1](#_ENREF_1)] | Automatic Flushing Area Identification | 6 concentrations (60, 20, 6.67, 2.22, 0.74, 0.25 mM) | 1min | 10 min  (every 10s) | Total Area | The total area of the flushing area over 10 min across all six niacin concentrations, calculated by an automated algorithm. |
| Degree | Karakula-Juchnowicz 2020[[8](#_ENREF_8)] | Automatic Flushing Area Identification | 3 concentrations (0.1, 0.01, 0.001 M) | 90s | 3, 5, 10, 15 min | Flushing area at 15 min for 0.01 M | The area of skin erythema was automatically calculated by an image processing algorithm at the 15th minute after application of 0.01 M niacin. |
| Degree | Qing 2022[[5](#_ENREF_5)] | Visual Semi-quantitative | 6 concentrations (60, 20, 6.67, 2.22, 0.74, 0.25 mM) | 1min | 10 min (every 10s) | Total VSQ Score | The sum of visual scores (0–3) across all time points and all six niacin concentrations. |
| Degree | Wang 2021[[7](#_ENREF_7)] (DD) | Visual Semi-quantitative | 4 concentrations (0.0001, 0.001, 0.01, 0.1 M) | 1min | 20 min (every 1 min) | Total VSQ Score | The sum of visual scores (0–3) across all time points and all four niacin concentrations. |
| Degree | Wang 2021[[7](#_ENREF_7)] (BD) | Visual Semi-quantitative | 4 concentrations (0.0001, 0.001, 0.01, 0.1 M) | 1min | 20 min (every 1 min) | Total VSQ Score | The sum of visual scores (0–3) across all time points and all four niacin concentrations. |
| Degree | Sun 2017[[9](#_ENREF_9)] | Visual Semi-quantitative | 4 concentrations (0.0001, 0.001, 0.01, 0.1 M) | 1min | 20 min (every 5 min) | Total VSQ Score | The sum of visual scores (0–3) across all time points and all four niacin concentrations. |
| Degree | Marouf 2016[[10](#_ENREF_10)] | Visual Semi-quantitative | 0.01 M, 0.1 M | 5 min | 20 min (every 5 min) | VSQ score at 15 min for 0.01 M | The visual score (0–3) assessed at the 15th minute after application of 0.01 M niacin. |
| Degree | Liu 2007[[13](#_ENREF_13)] | Visual Semi-quantitative | 3 concentrations (0.1, 0.01, 0.001 M) | 5 min | 15 min (every 5 min) | VSQ score at 15 min for 0.01 M | The visual score (0–3) assessed at the 15th minute after application of 0.01 M niacin. |
| Degree | Bosveld-van 2006[[14](#_ENREF_14)] | Visual Semi-quantitative | 0.1 M | 5 min | 30 s | VSQ score at 30 s for 0.1 M | The visual score (0–3) assessed at 30 s after application of 0.1 M niacin. |
| Degree | Ross 2004a[[15](#_ENREF_15)] | Visual Semi-quantitative | 5 concentrations (0, 0.1, 0.5, 1, 10 mM) | 1min | 15 min (every 5 min) | Total VSQ Score | The sum of visual scores (0–3) across all time points and all five niacin concentrations. |
| Degree | Maclean 2003[[17](#_ENREF_17)] | Visual Semi-quantitative | 4 concentrations (0.0001, 0.001, 0.01, 0.1 M) | 1min | 20 min (every 5 min) | Total VSQ Score | The sum of visual scores (0–3) across all time points and all four niacin concentrations. |
| Degree | Hu 2022[[4](#_ENREF_4)] | Laser Doppler Flowmetry | 8 concentrations (10^-5^, 10^-4^, 10^-3.5,^ 10^-3^, 10^-2.5^, 10^-2^, 10^-1.5^, 10^-1^ M) | 5min | 15 min | MBF | The highest BFR value observed across all eight niacin concentrations. |
| Degree | Gan 2022[[3](#_ENREF_3)] | Laser Doppler Flowmetry | 8 concentrations (10^-5^, 10^-4^, 10^-3.5,^ 10^-3^, 10^-2.5^, 10^-2^, 10^-1.5^, 10^-1^ M) | 5min | 15 min | MBF | The highest BFR value observed across all eight niacin concentrations. |
| Degree | Ma 2021[[6](#_ENREF_6)] | Laser Doppler Flowmetry | 8 concentrations (10^-5^, 10^-4^, 10^-3.5,^ 10^-3^, 10^-2.5^, 10^-2^, 10^-1.5^, 10^-1^ M) | 5min | 15 min | MBF | The highest BFR value observed across all eight niacin concentrations. |
| Degree | Yao 2016[[11](#_ENREF_11)] | Laser Doppler Flowmetry | 8 concentrations (10^-5^, 10^-4^, 10^-3.5,^ 10^-3^, 10^-2.5^, 10^-2^, 10^-1.5^, 10^-1^ M) | 5min | 15 min | MBF | The highest BFR value observed across all eight niacin concentrations. |
| Degree | Ross 2004b | Laser Doppler Flowmetry | 5 concentrations (0, 0.1, 0.5, 1, 10 mM) | 1min | 20 min | ΔF at 10 mM | Maximum plateau-phase increase in blood flow at 10 Mm. |
| Degree | Smesny 2010[[12](#_ENREF_12)] | Optical Reflectance Spectroscopy | 3 concentrations (0.1, 0.01, 0.001 M) | 90s | 15 min (every 3 min) | Oxyhemoglobin (HbO₂) | Skin flushing was quantified by the area under the curve of HbO₂ absorption. This was derived by subtracting pre-stimulation reflection intensities from test intensities and fitting Gaussian curves to the HbO₂ double peak. |
| Degree | Hudson 1997[[18](#_ENREF_18)] | Oral Niacin | 200 mg (oral) | NA | 45 min | Thermal Index (TI) | Vasodilation was quantified by a thermal index, calculated from changes in ear temperature relative to core body temperature and ambient room temperature. |
| Sensitivity | Hu 2022[[4](#_ENREF_4)] | Laser Doppler Flowmetry | 8 concentrations (10^-5^, 10^-4^, 10^-3.5,^ 10^-3^, 10^-2.5^, 10^-2^, 10^-1.5^, 10^-1^ M) | 5min | 15 min | lgEC₅₀ (dose) | The log-transformed half-maximal effective concentration. It was calculated by fitting a dose-response curve to the BFR values obtained across eight niacin concentrations. |
| Sensitivity | Ma 2021[[6](#_ENREF_6)] | Laser Doppler Flowmetry | 8 concentrations (10^-5^, 10^-4^, 10^-3.5,^ 10^-3^, 10^-2.5^, 10^-2^, 10^-1.5^, 10^-1^ M) | 5min | 15 min | lgEC₅₀ (dose) | The log-transformed half-maximal effective concentration. It was calculated by fitting a dose-response curve to the BFR values obtained across eight niacin concentrations. |
| Sensitivity | Yao 2016[[11](#_ENREF_11)] | Laser Doppler Flowmetry | 8 concentrations (10^-5^, 10^-4^, 10^-3.5,^ 10^-3^, 10^-2.5^, 10^-2^, 10^-1.5^, 10^-1^ M) | 5min | 15 min | EC₅₀ (dose) | The half-maximal effective concentration. It was calculated by fitting a dose-response curve to the BFR values obtained across eight niacin concentrations. |
| Speed | Wang 2023[[2](#_ENREF_2)] | Automatic Flushing Area Identification | 6 concentrations (60, 20, 6.67, 2.22, 0.74, 0.25 mM) | 1min | 10 min (every 10s) | EC₅₀ (time) | The time required to reach 50% of the maximum flushing area at the highest concentration (60 mM), calculated by nonlinear fitting of the time-response curve. |
| Speed | Wang 2021[[7](#_ENREF_7)] (DD) | Visual Semi-quantitative | 4 concentrations (0.0001, 0.001, 0.01, 0.1 M) | 1min | 20 min (every 1 min) | EC₅₀ (time) | The time required to reach 50% of the maximum flushing score at the highest concentration (0.1 M), derived from nonlinear fitting of the time-response curve. |
| Speed | Wang 2021[[7](#_ENREF_7)] (BD) | Visual Semi-quantitative | 4 concentrations (0.0001, 0.001, 0.01, 0.1 M) | 1min | 20 min (every 1 min) | EC₅₀ (time) | The time required to reach 50% of the maximum flushing score at the highest concentration (0.1 M), derived from nonlinear fitting of the time-response curve. |

DD: Depressive disorder; MBF: Maximal blood flow; BD: Bipolar disorder; VSQ: Visual semi-quantitative method.

Blood Flow Response (BFR) is defined as the increase in skin blood flow following niacin stimulation, calculated by subtracting the pre-stimulation blood flow value from the post-stimulation value.

Visual score (0–3): A semi-quantitative assessment of erythema degree, manually scored by raters. The response is graded as follows: 0 = no visible erythema; 1 = minimal erythema, typically within the stimulated area; 2 = moderate erythema, covering the entire stimulated area; 3 = maximal erythema, extending beyond the stimulated area.


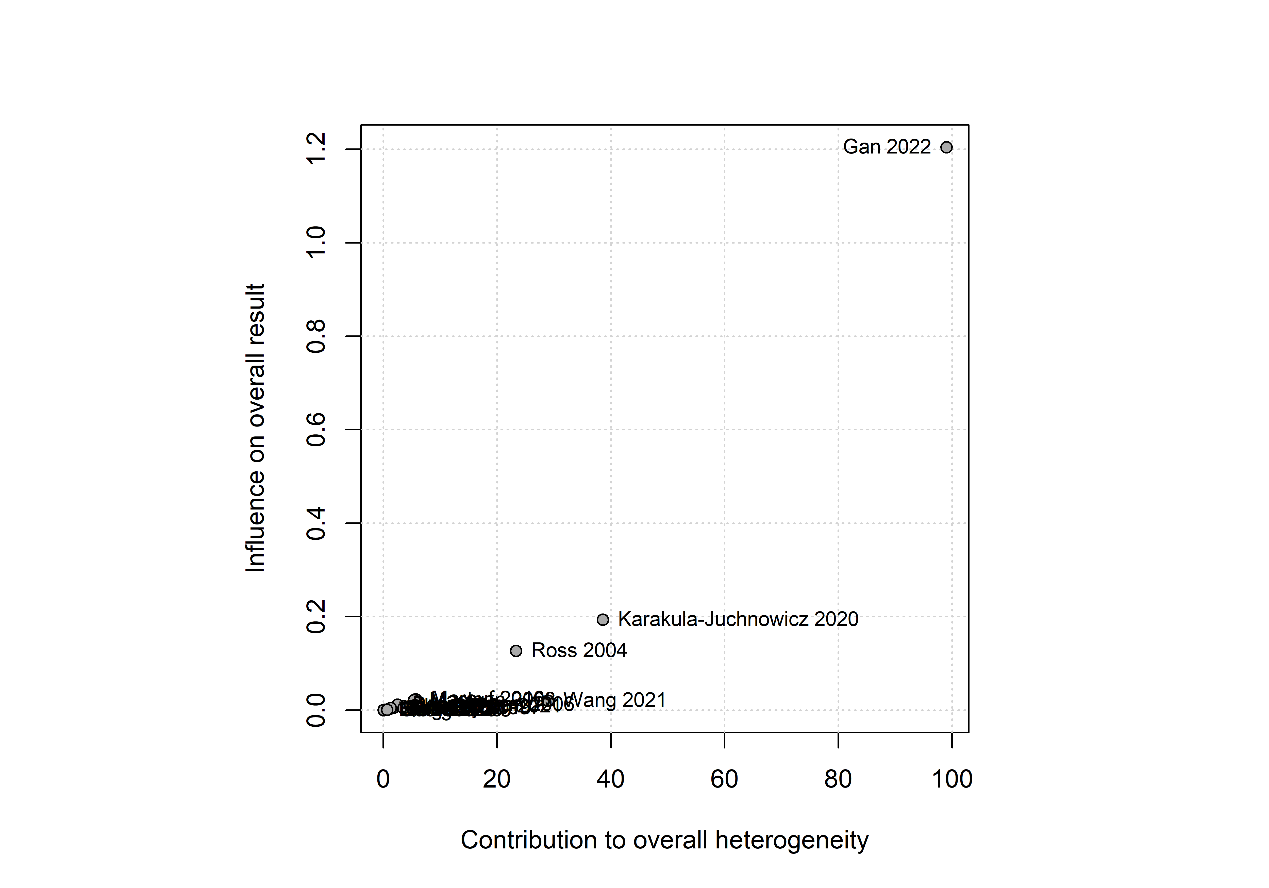


**Supplementary Figure 1. The Baujat plot of the degree of the niacin skin flushing response in mood disorders compared to healthy controls.**


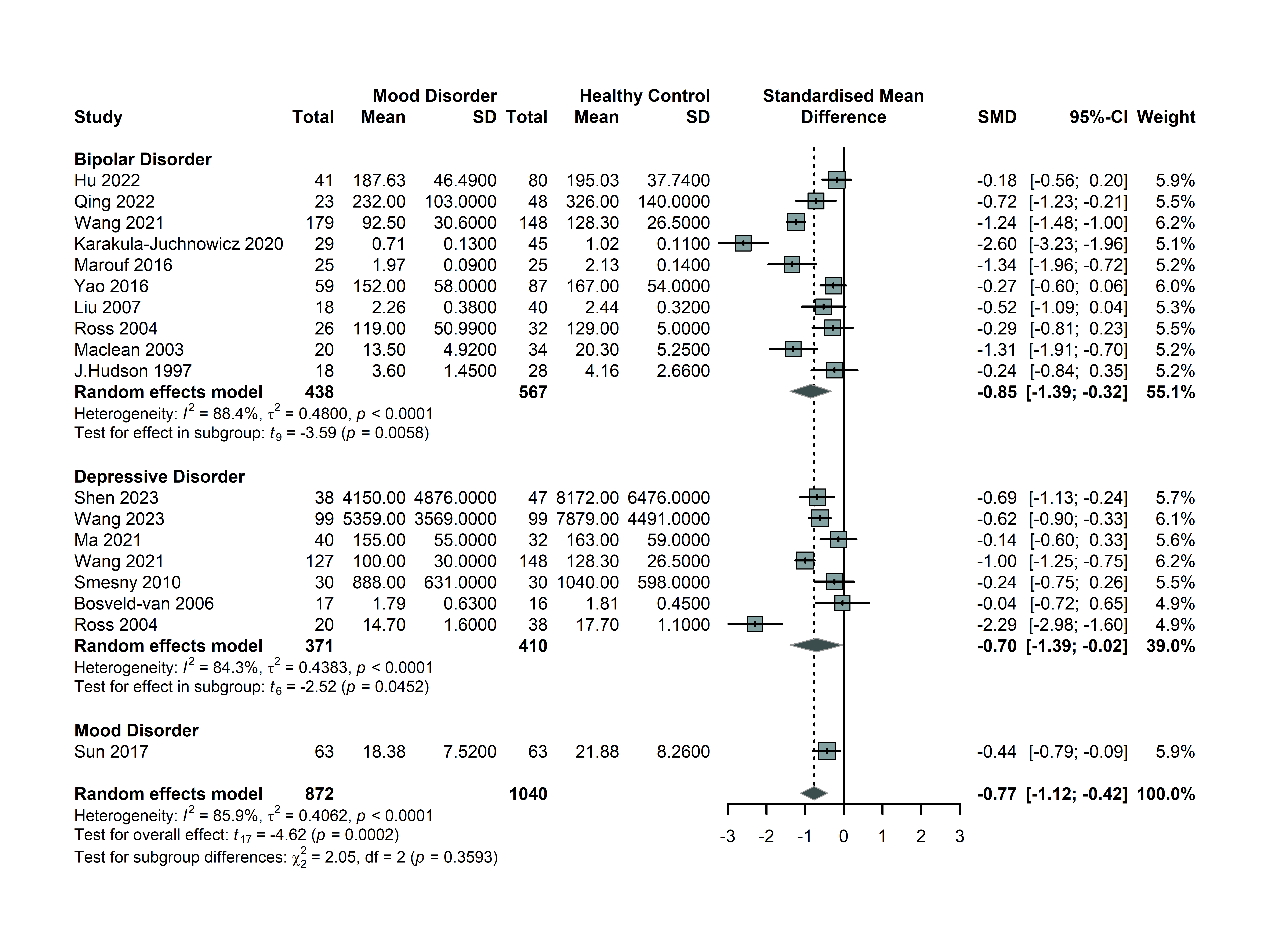


**Supplementary Figure 2. Forest plot of** **the degree of the niacin skin flushing response in mood disorders compared to healthy controls (Excluding Gan 2022).** SD: Standard Deviation; SMD: Standardized Mean Difference; CI: Confidence Interval.


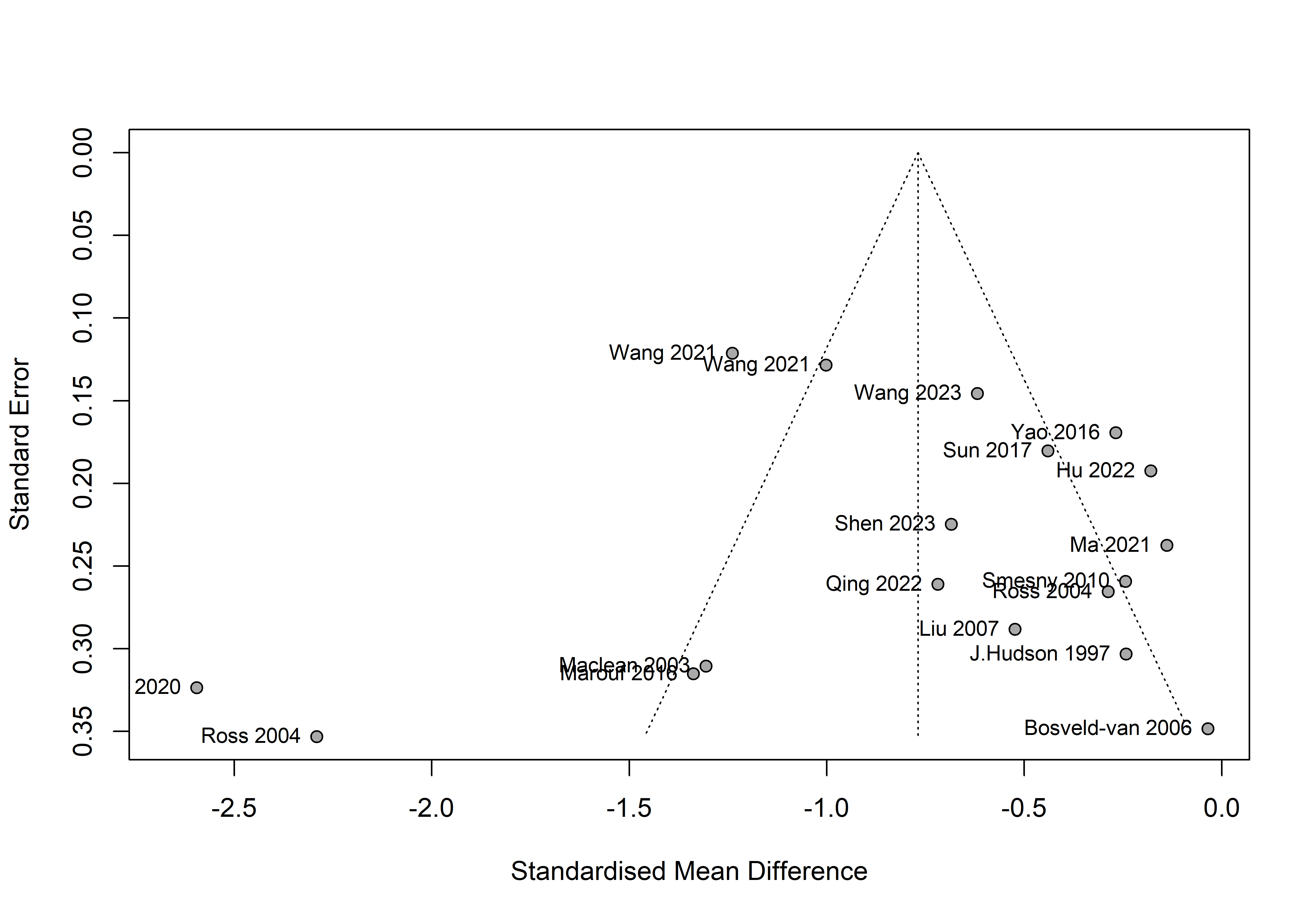


**Supplementary Figure 3. Funnel plots for identifying publication bias in the meta-analysis of the degree of the niacin skin flushing response.**


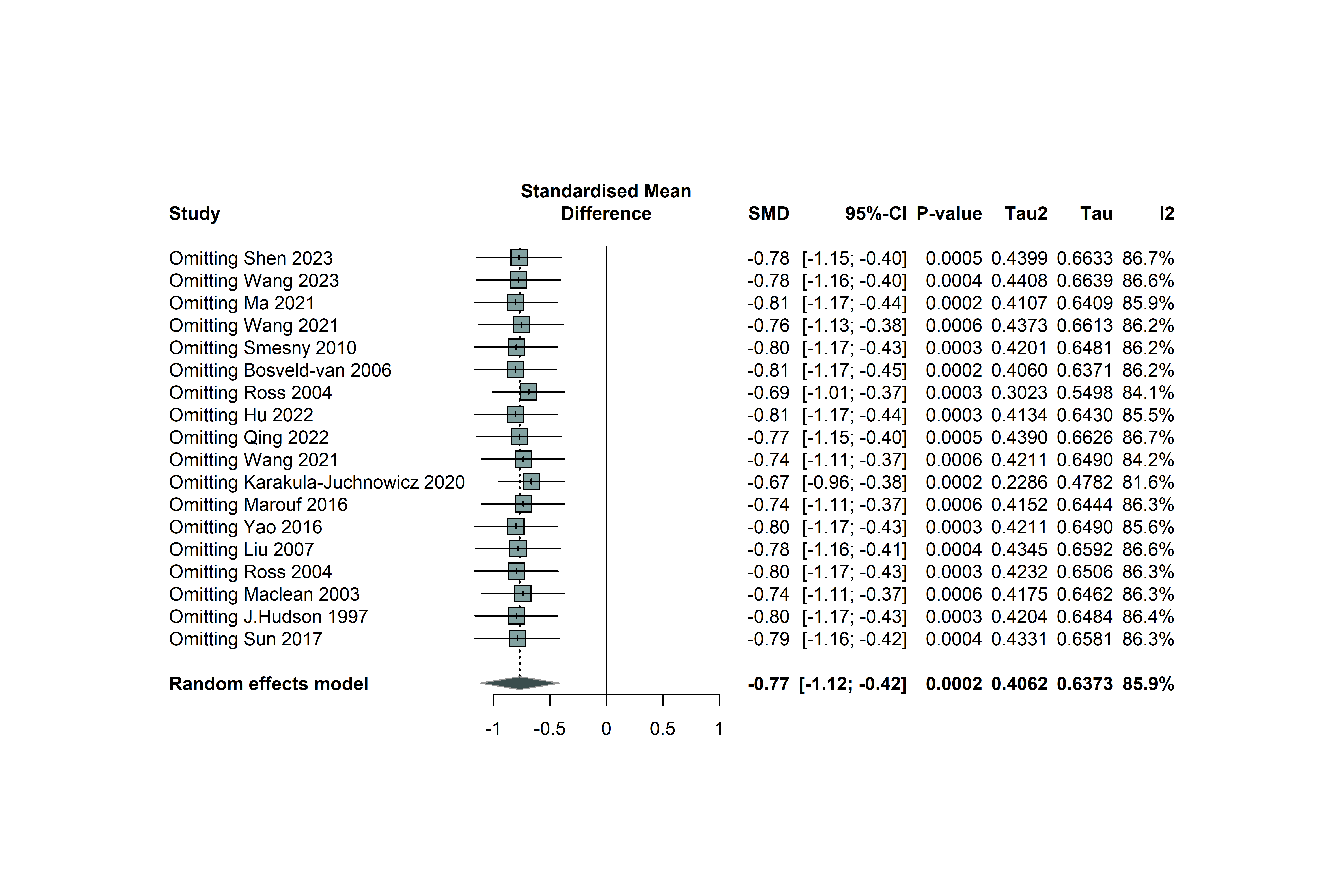


**Supplementary Figure 4. The leave-one-out sensitivity analysis of the degree of the niacin skin flushing response.** SMD: Standardized Mean Difference; CI: Confidence Interval.

**References**

1. Nianhong S, Pan L, Caijun L, et al., "Diagnostic value of niacin skin blunting response in adolescent patients with depression," BMC Psychiatry 23 (2023):

2. Wang J, Qing Y, Liang J, et al., "Identification of adolescent patients with depression via assessment of the niacin skin flushing response," Journal of Affective Disorders 324 (2023): 69-76

3. Gan R, Zhao Y, Wu G, et al., "Replication of the abnormal niacin response in first episode psychosis measured using laser doppler flowmeter," Asia Pac Psychiatry 14 (2022): e12516

4. Hu Y, Xu L, Gan R, et al., "A potential objective marker in first-episode schizophrenia based on abnormal niacin response," Schizophrenia Research 243 (2022): 405-412

5. Qing Y, Liang J, Wang J, et al., "Attenuated niacin skin flushing response in children and adolescents with mental disorders: A transdiagnostic early warning marker," Schizophrenia Research 248 (2022): 32-34

6. Ruihua M, Yang L, Panqi L, et al., "Correlation between niacin skin flushing and cognitive function in patients with depression," Chinese Journal of Psychiatry 54 (2021): 204-210

7. Wang DD, Hu XW, Jiang J, et al., "Attenuated and delayed niacin skin flushing in schizophrenia and affective disorders: A potential clinical auxiliary diagnostic marker," Schizophrenia Research 230 (2021): 53-60

8. Karakula-Juchnowicz H, Rog J, Wolszczak P, et al., "Skinrems—a new method for assessment of the niacin skin flush test response in schizophrenia," Journal of Clinical Medicine 9 (2020): 1-16

9. Sun L, Yang X, Jiang J, et al., "Identification of the niacin-blunted subgroup of schizophrenia patients from mood disorders and healthy individuals in chinese population," Schizophrenia Bulletin 44 (2018): 896-907

10. Maroufi M, Tabatabaeian M, Tabatabaeian M, et al., "Comparison of niacin skin flush response in patients with schizophrenia and bipolar disorder," Iranian Journal of Psychiatry and Behavioral Sciences 10 (2016):

11. Yao JK, Dougherty GG, Gautier CH, et al., "Prevalence and specificity of the abnormal niacin response: A potential endophenotype marker in schizophrenia," Schizophrenia Bulletin 42 (2016): 369-376

12. Smesny S, Baur K, Rudolph N, et al., "Alterations of niacin skin sensitivity in recurrent unipolar depressive disorder," Journal of Affective Disorders 124 (2010): 335-340

13. Liu CM, Chang SS, Liao SC, et al., "Absent response to niacin skin patch is specific to schizophrenia and independent of smoking," Psychiatry Research 152 (2007): 181-187

14. Bosveld-van Haandel L, Knegtering R, Kluiter H, et al., "Niacin skin flushing in schizophrenic and depressed patients and healthy controls," Psychiatry Research 143 (2006): 303-306

15. Ross BM, Ward P, Glen I, "Delayed vasodilatory response to methylnicotinate in patients with unipolar depressive disorder," Journal of Affective Disorders 82 (2004): 285-290

16. Ross BM, Hughes B, Turenne S, et al., "Reduced vasodilatory response to methylnicotinate in schizophrenia as assessed by laser doppler flowmetry," European Neuropsychopharmacology 14 (2004): 191-197

17. Maclean R, Ward PE, Glen I, et al., "On the relationship between methylnicotinate-induced skin flush and fatty acids levels in acute psychosis," Progress in Neuro-Psychopharmacology and Biological Psychiatry 27 (2003): 927-933

18. Hudson CJ, Lin A, Cogan S, et al., "The niacin challenge test: Clinical manifestation of altered transmembrane signal transduction in schizophrenia?," Biological Psychiatry 41 (1997): 507-513
